# Sense of Community Mediating Between Age-Friendly Characteristics and Life Satisfaction of Community-Dwelling Older Adults

**DOI:** 10.3389/fpsyg.2020.00086

**Published:** 2020-03-04

**Authors:** Alma Au, Daniel W. L. Lai, Ho-ming Yip, Stephen Chan, Simon Lai, Habib Chaudhury, Andrew Scharlach, George Leeson

**Affiliations:** ^1^Department of Applied Social Sciences, The Hong Kong Polytechnic University, Kowloon, Hong Kong; ^2^Department of Gerontology, Simon Fraser University, Burnaby, BC, Canada; ^3^School of Social Welfare, University of California, Berkeley, Berkeley, CA, United States; ^4^Oxford Institute of Population Ageing, University of Oxford, Oxford, United Kingdom

**Keywords:** successful aging, social participation, community health support, sustainability, population aging

## Abstract

The growth of age-friendly community initiatives underscores a paradigmatic shift from the individual to the community, addressing dynamic transactions between people and the environment they are living in. The purpose of the present study is to address the gap in existing research by examining the psycho-social effects of the sense of community in mediating between WHO domains of age-friendliness and the life satisfaction of older adults. Data were obtained from 898 participants in Hong Kong. Path analysis was conducted. Two AFC domains, Social Participation as well as Community Support and Health Services, were found to be associated with life satisfaction. Sense of community was found to mediate between these two domains and life satisfaction. The implications of these findings are discussed with reference to developing opportunities in social participation of older adults and enhancing community/health support services in the context of developing sustainability in the community.

## Introduction

The composition of local communities and wider societies is rapidly changing, as population aging increases at an unprecedented rate. According to the World Health Organization (World Health Organization [WHO], 2015a), the proportion of the world’s population over 60 years will nearly double from 12 to 22% between 2015 and 2050. The older population is growing faster in urban areas than in rural areas. According to the United Nations (2017), between 2000 and 2015, the global number of people aged 60 years or over increased by 68% in urban areas, compared to a 25% increase in rural areas. As a result, older adults are increasingly concentrated in urban areas, with 58% of the world population aged 60 years or over residing in urban centers. In 2050, 80% of older people will be living in low- and middle-income countries. China’s population is aging at a faster rate than almost all other countries (United Nations, 2017). Hong Kong is part of China, but it also has a unique colonial history and “East-meet-West” culture. Waves of migration since the turn of the century, triggered by political upheavals and economic developments, have shaped Hong Kong’s population demographics (Welsh, 1993; Lim, 2002). According to the Hong Kong Population Projections for 2012 to 2041, the proportion of the population aged 65 and over will rise from 13% in 2011 to 30% in 2041 (Census and Statistics Department, 2017). The life expectancy in Hong Kong has topped the worldwide rankings (United Nations, 2017).

The World Health Organization [WHO] (2007) refers specifically to enhancing the age-friendliness of urban centers, calling for “a necessary and logical response to promote the wellbeing and contributions of older urban residents and keep cities thriving” (p. 4). Urban environments can affect older adult’s well-being due to concerns regarding safety and security, health risks, access to housing and services, and social isolation and exclusion (Barusch, 2013; Buffel and Phillipson, 2016). The aging population and urbanization trends illustrate the importance of considering the ways in which “age-friendly” urban planning can enhance the well-being of older adults, linked to concepts of successful and active aging. Successful aging can be understood as involving avoiding disease and disability and maintaining high cognitive and physical functioning and engagement with life (Rowe and Kahn, 1997). The WHO’s Active Aging Policy Framework focuses on the concept of “active aging”, which is defined as a process of optimizing opportunities for health, participation, and security in order to enhance people’s quality of life as they age. This enhances people’s ability to realize physical, social, and mental well-being over the life course, including access to adequate protection, security, and care. Efforts to develop Hong Kong into an age-friendly city were stated the 2016 Policy Address. The importance of promoting active aging has been reinforced as the new generation of older adults will be healthier, more energetic, better educated, and capable of making further contributions to the community (Chief Executive of Hong Kong Special Administrative Region, 2016).

While ecological frameworks that consider the influence of environment on development have a long history (Lawton and Nahemow, 1973), the rapid growth of age-friendly community (AFC) initiatives in recent years suggest a rather unique approach focused on deliberately influencing social and physical environments to benefit older adults (World Health Organization [WHO], 2007). These initiatives reflect a paradigmatic shift from the individual to the community, addressing dynamic transactions between people and their environment. Reviews of active and successful aging have highlighted the need to view individual development as a dynamic life-long process, influenced by time and place and embedded in the web of relationships and social structures (Stowe and Cooney, 2015). Apart from physical attributes, the cultivation of place is shaped by individual life experience and intentions as well as social participation and shared expectations (Moore and Ekerdt, 2011). Aging is thus not only a physical process but also a psycho-social process embedded in the community and cultural setting (Li and Au, 2019). In a study comparing the results of a WHO AFC questionnaire in two districts with different infrastructure levels, it was observed that satisfaction levels in AFC domains were not solely dependent on amenities (Wong et al., 2015). These findings warrant further research on psychosocial factors that may influence residents’ perceptions of local environments. The purpose of the present study is to address this research gap by examining the effects of the sense of community in mediating between WHO domains of age-friendliness and the life satisfaction of older adults.

“Age-friendly” communities include environmental as well as social features, which are both necessary to ensure older adults’ well-being and participation (Scharlach and Lehning, 2013; Fitzgerald and Caro, 2014; Steels, 2015). International studies of older adults have reported that quality of life (including subjective well-being and happiness, social connections, and health) is affected by factors such as built environment (e.g., amenities, transportation, and parks), neighborhood conditions, and government and community services (Plouffe and Kalache, 2010; Yip et al., 2013; Hogan et al., 2016; Park and Lee, 2017). Environmental characteristics in the home and the wider neighborhood have multi-level significance for old age and the aging society (Woo et al., 2017; Chaudhury and Oswald, 2018). The World Health Organization [WHO] (2007) has identified eight domains as key aspects of AFCs. These include outdoor spaces and buildings, housing, transportation, communication and information, community support and health services, respect and inclusion, social participation, and civic participation and employment (Evans and Stoddart, 1990; Feldman and Oberlink, 2003).

AFCs should facilitate a medium for the unfolding of both personal and environmental pathways to promote well-being thought life-course developments through the older adults’ connection and contributing to the community (Scharlach, 2017). Wenner and Randall (2016) has identified that a sense of community cohesion was predictive of prosocial behavior for middle-aged and older adults. Social participation has also been found to be associated with life satisfaction in both young–old and old–old adults in Hong Kong (Au et al., 2017). Wenner and Randall (2016) further noted that prosocial behavior can have generative dimension as caring behaviors can often benefit the next generation. Au et al. (2019b) have found significant associations between generative behavior and positive emotions in older adults across three countries. Thus, aging-in-place has implications for life satisfaction not only for the older adults but also for the next generation (Au et al., 2019a; Oswald et al., 2011). The concept of the sense of community can provide a way of capturing the mechanism of change involved in building a more age-friendly community.

Sense of community can be conceptualized along two dimensions: territorial and relational (Mak et al., 2009). While the territorial element refers to identification with a shared geographical location, the relational element refers to the quality of social relationships. McMillan and Chavis (1986) identified four possible components of sense of community: membership, influence, reinforcement, and shared emotional connection. Previous international studies have found that among the wider population, sense of community, community connections, and attachment to one’s place of residence are significantly correlated with community participation, social support, prosocial behavior, psychological empowerment, and mental health (Cicognani et al., 2008; Peterson et al., 2008; Mak et al., 2009; Stanley et al., 2010; Speer et al., 2013; Talò et al., 2014) and with general quality of life (Rollero and De Piccoli, 2010; Gattino et al., 2013; Tartaglia, 2013). Studies have also reported that sense of community or social connectedness can mediate the relationship between external environments and active aging and well-being among older adults (Bess et al., 2002; Nowell and Boyd, 2010; Gardner, 2011; Lai et al., 2016; Sun et al., 2017).

Older adults commonly identify community participation and engagement, social interactions and connectedness, and “neighborliness” as central to AFCs (Feldman and Oberlink, 2003; Emlet and Moceri, 2012; Cho and Kim, 2016; Shank and Cutchin, 2016). As it is associated with psychological well-being and resilience, sense of community can have particular relevance for collectivistic cultures experiencing transformation, such as Chinese societies undergoing rapid urban development (Chao and Huang, 2016; Zhang et al., 2017). Mak et al. (2009) found that sense of community in Hong Kong was negatively associated with daily hassles and positively with social support. Yip et al. (2013) found that while neighboring behaviors enhanced subjective well-being of the young and middle-aged residents, it has no impact on older people in urban China. On the other hand, the sense of community was found to contribute to well-being of all age groups and was particularly strong among older people in more deprived neighborhoods.

The psycho-social relevance of the sense of community has not been extensively studied in Chinese communities in relation to age-friendly city initiatives. The objective of the present study is to address this gap in existing research by examining the role of sense of community in mediating the effects of age-friendliness (based on WHO domains) on life satisfaction in Hong Kong’s aging population. To address this objective, three research questions will be examined:

1. What is the effect of the perceived age-friendliness characteristics on life satisfaction among aging people in the community?
2. What is the effect of the perceived age-friendliness characteristics on sense of community among aging people in the community?
3. Does the sense of community play a mediating role between the effects of the perceived aged-friendliness characteristics and life satisfaction among aging people in the community?

## Materials and Methods

### Procedures and Participants

Questionnaires were used to collect data from a variety of settings including community centers, public area in housing estates, shopping areas, and parks. The administration of questionnaire took about 30 to 40 min. The data were collected from two regions in Kowloon East (Kowloon City District Council, 2015; Kwun Tong District Council, 2015) using a convenience sampling approach. Data collection took place from January 2016 to April 2016. Recruitment focused on adults aged above 55 with intact hearing and no mental health challenges.

### Measurements

#### Socio-Demographic Variables

Socio-demographic variables collected in this study included age, sex, education level, subjective health, marital status, and income. They were treated as covariates in the analyses. Subjective health was measured using five-point Likert-type scales, ranging from 1 (“bad”) to 5 (“excellent”) (Yu and Moses et al., 2018). Financial satisfaction was measured using a five-point Likert scale from 1 (“very insufficient”) to 5 (“very sufficient”) (Hira and Mugenda, 1998; Garrett and James, 2013). They were treated as covariates in the analyses. These variables were associated with life satisfaction in previous studies (Rentfrow et al., 2009; Swift et al., 2014; Macia et al., 2015).

#### The Age-Friendly City Scale (AFC)

The indicators of age-friendliness in cities (World Health Organization [WHO], 2015b) were adapted into a structured questionnaire and translated into Chinese (Wong et al., 2015). Eight domains were assessed using 53 items. The constructs have been validated by both qualitative and quantitative methods (World Health Organization [WHO], 2015a, b; Lai et al., 2016). Participants rated their views on each item using a six-point Likert-type scale ranging from 1 (“strongly disagree”) to 6 (“strongly agree”). Responses for each domain were averaged as a mean score to represent the level of perceived “friendliness” in each domain. The reliability (Cronbach’s alpha) estimates were 0.76 for Outdoor Spaces and Buildings (e.g., pavements are well-maintained, free of obstructions and reserved for pedestrians), 0.85 for Transportation (e.g., public transportation costs are affordable), 0.71 for Housing (e.g., sufficient and affordable home maintenance and support services are available), 0.81 for Social Participation (e.g., a wide variety of activities is offered to appeal to a diverse population of older people), 0.77 for Respect and Social Inclusion (e.g., older people are recognized by the community for their past as well as their present contributions), 0.75 for Civic Participation and Employment (e.g., a range of flexible and appropriately paid opportunities for older people to work is promoted), 0.75 for Communication and Information (e.g., an effective communication system reaches community residents of all ages), and 0.71 for Community Support and Health Services (e.g., an adequate range of support services is offered).

#### The Brief Sense of Community Scale (SOC)

The SOC is an eight-item scale that uses the McMillan and Chavis (1986) model to assess dimensions of needs fulfillment, group membership, influence, and emotional connection (Peterson et al., 2008). Items were rated on a five-point Likert-type scale ranging from “strongly disagree” to “strongly agree” to describe a participant’s community experiences. A high score on the SOC indicated a better sense of community. This measure demonstrated good psychometric properties for Chinese participants (Li et al., 2011; Wu and Chow, 2013; Huang and Wong, 2014). In this study, Cronbach’s alpha of the overall scale was 0.80.

#### The Satisfaction With Life Scale (SWLS)

Satisfaction with life was measured by the SWLS, which evaluates the cognitive component of subjective well-being (Diener et al., 1985). It consists of five items using a seven-point Likert-type scale ranging from 1 (“strongly disagree”) to 7 (“strongly agree”). The mean score of this scale was used for analysis, with a higher score representing a higher level of satisfaction. The reliability of SWLS was 0.89 in the current study.

### Procedures

The protocol was approved by the Research Ethics Committee of the Hong Kong Polytechnic University (2016). All subjects gave written informed consent in accordance with the Declaration of Helsinki. Trained research helpers introduced the background of the study to the participants. Participants then completed a set of self-administered questionnaires after signing an informed consent form. Research helpers assisted a few aged participants who had difficulties in reading or writing to complete the questionnaire by reading out the questions and writing down their responses to the items.

### Statistical Analyses

Descriptive statistics for the overall sample and for the two districts, Kowloon City and Kwun Tong, were calculated for the following items: (1) Eight domains of AFC Scales, (2) SWLS, and (3) the sense of community as measured by SOC. Correlations were calculated to understand the relationship among AFC domains, SOC, and SWLS, which also informed the multiple regression analyses. Multiple regression analyses were performed with SWLS as the outcome variable and AFC domains as the predictors, to understand which AFC domains were unique predictors of SWLS. To specify on which domain of AFC could be contributing to SWLS, we first use the enter method for all socio-demographic variables and then a stepwise approach of recruiting eight AFC domains into the models. The next task was to examine the mediating role of SOC in the relationship of AFC domains with SWLS. The association of AFC domains with SOC was established and tested with multiple regression analyses using SOC as the outcome variable and AFC domains that significantly predicted SWLS as the predictors. After these procedures, AFC domains that were independent predictors for both SWLS and SOC in the overall sample were entered as multiple predictors in the mediation model.

The multiple predictors mediation model included the chosen AFC domains as predictor variables, SOC as the mediating variable, and SWLS as the outcome variable. Socio-demographic variables were treated as covariates in all regression models. The PROCESS macro provided by Hayes (2013) was used for mediation analyses. Bias-corrected and accelerated 95% confidence intervals (BCa 95% CI) of the indirect effects (*ab*) were estimated using 10000 bootstrap sample (Efron and Tibshirani, 1994). Total effect (*c*) and direct effect (*c*′) were also reported. Of note, mediating variables with BCa 95% CI that did not contain zero were considered statistically significant (Hayes, 2013).

## Results

### Descriptive Statistics

The sample of 898 older adults were recruited from Kowloon City (*n* = 430; 47.88%) and Kwun Tong (*n* = 468; 52.12%). Around 90% of the participants were recruited from community centers and 10% were recruited from public areas including shopping areas and parks. Demographic information for the participants is provided in Table 1. The mean age of the overall sample was 70.71 (*SD* = 8.73), ranging from 55 to 97. There were 263 men (29.29%) and 635 women (70.71%). The correlations between all the eight AFC domains with SOC and with SWLS were significant (*p* < 0.05) (Table 2).

**TABLE 1:** Descriptive statistics.

	**Overall (*N* = 898)**
	***M* (*SD*)**
**Socio-demographic variables**	
Age	70.71 (8.73)
Gender (Male/Female)	263/635
Marital status (Married/Not married)	510/388
Income	7869.26 (5809.51)
Education	
Primary or below	486
Secondary	341
Post-secondary	71
Employment (Yes/No)	(61/837)
Subjective health	2.38 (0.94)
Financial satisfaction	3.08 (0.69)
**AFC domains**	
Outdoor spaces and buildings	4.07 (0.71)
Transportation	4.38 (0.62)
Housing	3.97 (0.96)
Social participation	4.57 (0.71)
Respect and social inclusion	4.23 (0.79)
Civic participation and employment	4.04 (0.91)
Communication and information	4.17 (0.77)
Community support and health services	3.89 (0.77)
Sense of community	3.82 (0.47)
Satisfaction with life	4.67 (1.01)

**TABLE 2:** Zero-order correlations between AFC domains, sense of community, and satisfaction with life.

	**1**	**2**	**3**	**4**	**5**	**6**	**7**	**8**	**9**	**10**
Outdoor Spaces and Buildings	1	0.65**	0.44**	0.41**	0.51**	0.38**	0.42**	0.48**	0.34**	0.29**
Transportation		1	0.44**	0.46**	0.52**	0.38**	0.40**	0.55**	0.36**	0.33**
Housing			1	0.47**	0.43**	0.32**	0.40**	0.43**	0.28**	0.30**
Social participation				1	0.57**	0.42**	0.48**	0.44**	0.43**	0.37**
Respect and social inclusion					1	0.53**	0.53**	0.52**	0.37**	0.28**
Civic participation and employment						1	0.45**	0.39**	0.35**	0.23**
Communication and information							1	0.48**	0.34**	0.22**
Community support and health services								1	0.38**	0.34**
Sense of community									1	0.48**
Satisfaction with life										1

*p < 0.05; **p < 0.01.

### Multiple Regression of SWLS on AFC Domains

Satisfaction with life scale was first regressed on socio-demographic variables. It was found that age, financial satisfaction, and health status were associated with SWLS. After controlling for socio-demographic variables, only Social Participation and Community/Health Services were found to be the significant predictors of SWLS in Model 2 using the stepwise approach; similar results were identified by using the enter method (Table 3).

**TABLE 3:** Multiple regression of Satisfaction with Life on AFC domains and socio-demographic variables for the overall sample.

	**Model 1**				**Model 2**			
	***B***	***SE***	**β**	***p***	***B***	***SE***	**β**	***p***
Age	0.02	0.00	0.21	<0.01	0.02	0.00	0.14	<0.01
Gender	0.03	0.07	0.01	0.71	−0.02	0.07	−0.01	0.75
Marital status	0.04	0.06	0.02	0.57	0.01	0.06	0.00	0.91
Income	0.02	0.01	0.05	0.18	0.03	0.01	0.06	0.07
Education	−0.07	0.02	−0.11	<0.01	−0.03	0.02	−0.06	0.08
Employment	−0.03	0.13	−0.01	0.76	0.02	0.12	0.01	0.84
Subjective health	0.24	0.03	0.22	<0.01	0.20	0.03	0.19	<0.01
Financial satisfaction	0.46	0.05	0.31	<0.01	0.40	0.05	0.27	<0.01
Social participation					0.30	0.05	0.21	<0.01
Community support and health services					0.19	0.05	0.14	<0.01
*R*^2^			0.23				0.34	
*F* for change in *R*^2^			32.21	<0.01			28.46	<0.01

### Multiple Regression of SOC on AFC Domains

Sense of community was first regressed on the socio-demographic variables followed by eight domains of AFC by using a similar approach. Results indicated that similar socio-demographic variables were associated with SOC. Moreover, four AFC domains, namely, Social Participation, Community/Health Services, Civic Participation and Employment, and Outdoor Spaces and Buildings, were associated with SOC after controlling socio-demographic variables using stepwise regression. Furthermore, Social Participation, Community/Health Services, and Civic Participation and Employment were found to be the strongest predictors of SOC after controlling socio-demographic variables using the stepwise approach; the remaining factor regarding Outdoor Spaces and Buildings did not explain the higher variances in the model. We then conducted another multiple regression model using the enter method for prediction of SOC by controlling socio-demographic variables. These three domains significantly predicted SOC with socio-demographic covariates adjusted (*p* < 0.01) (Table 4). Since only Social Participation and Community/Health Services were commonly associated with both SOC and SWLS after controlling covariates, the pre-requisite for studying SOC as a mediator in the relationship of AFC domains with SWLS was fulfilled.

**TABLE 4:** Multiple regression of Sense of Community on AFC domains and socio-demographic variables for the overall sample.

	**Model 1**				**Model 2**			
	***B***	***SE***	**β**	***p***	***B***	***SE***	**β**	***p***
Age	0.01	0.00	0.09	0.02	0.00	0.00	0.00	0.94
Gender	0.03	0.04	0.02	0.49	−0.01	0.03	−0.01	0.87
Marital status	0.01	0.03	0.01	0.75	−0.01	0.03	−0.01	0.69
Income	−0.01	0.01	−0.02	0.52	0.00	0.01	0.00	0.87
Education	−0.01	0.01	−0.05	0.17	0.01	0.01	0.02	0.52
Employment	0.12	0.07	0.01	0.62	0.10	0.06	0.06	0.10
Subjective health	0.04	0.02	0.08	0.02	0.02	0.02	0.03	0.30
Financial satisfaction	0.14	0.03	0.21	<0.01	0.11	0.03	0.23	<0.01
Social participation					0.15	0.05	0.21	<0.01
Community support and health services					0.08	0.02	0.14	<0.01
Civic participation and employment					0.06	0.02	0.11	<0.01
*R*^2^			0.07				0.29	
*F* for change in *R*^2^			8.21	< 0.01			22.50	< 0.01

### Mediation Analyses of SOC in the Association of AFC Domains With SWLS

**FIGURE 1:**
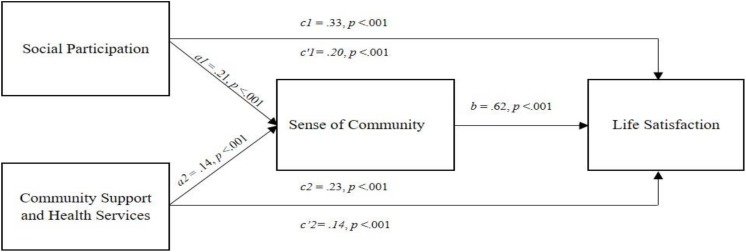
Path diagram of the relations between AFC domains (Social Participation, Community Support and Health Services), Sense of Community, and Satisfaction with Life for the overall sample with *a*, *b*, *c*, and *c*′ denoting regression coefficients, *c* denoting total effect, and *c*′ denoting direct effect.

Two AFC domains, Social Participation and Community/Health Services, were entered in the multiple predictor mediation model as independent variables and they were significant predictors of both SWLS and SOC. The results are shown in Table 5. Social Participation had a total effect on SWLS, *c* = 0.33, *p* < 0.001, which was the sum of an indirect effect on SWLS through SOC (*ab* = 0.13, BCa 95% CI [0.09, 0.17]) and a direct effect on SWLS (*c*′ = 0.20, *p* < 0.001). Community/Health Services had a total effect on SWLS (*c* = 0.23, *p* < 0.001), which included an indirect effect on SWLS through SOC (*ab* = 0.09, BCa 95% CI [0.06, 0.12]) and a direct effect on SWLS (*c*′ = 0.14, *p* < 0.001). The mediating role of SOC in the effects of the two AFC domains, Social Participation and Community Health Services, on SWLS was supported (Figure 1).

**TABLE 5:** Multiple predictor mediation from AFC domains to satisfaction with life through sense of community. Age, educational level, subjective expenditure, subjective health, income, and marital status (married vs. others) were controlled for.

	**Total effect**		**Effect size**	**Direct effect**		**Effect size**	**Indirect effect**	**Effect size**	**Ratio of**
	***c***	***p***	***c*′*cs***	***c*′**	***p***	***c*′*cs***	***ab* [BCa 95% CI]**	***c*′*cs***	**indirect effect to total effect**
**Overall (*N* = 898)**									
Social participation	0.33	<0.001	0.23	0.20	<0.001	0.14	0.13 [0.09, 0.17]	0.09	0.39
Community support and health services	0.23	<0.001	0.17	0.14	<0.001	0.11	0.09 [0.06, 0.12]	0.07	0.38

## Discussion

The present study enhances our understanding of how social participation and community/health support services contribute to the life satisfaction of older adults through the mediating effects of sense of community. The importance of social participation on life satisfaction has also been documented by Li et al. (2018) in China. At the same time, the happiness of older adults has been found to relate to the quality of services that enable resident to age in place (Hogan et al., 2016). The importance of place attachment and identity has been highlighted and has been discussed by Mak et al. (2009). While resonating findings from previous work, the present study has contributed to a more comprehensive view of highlighting the significance of both social participation and community/health services in contributing to the life satisfaction of older adults with the sense of community as the mediator.

Results from the study would support the positive association between community engagement and well-being in older adults (Scharlach, 2017). While the connections between urban environments and the sense of community has been noted (Francis et al., 2012; Zhang and Lin, 2012), the present study highlights the relevance of making socially inclusive and supportive environments to enable older adults to meet age-related requirements in order to continue living in their homes and communities for as long as they choose to. These enhancements should therefore involve sustaining opportunities for developing and maintaining significant relationships, participating in the community in ways meaningful to them and access to community and health services (Chaudhury et al., 2012; Wahl et al., 2012; Scharlach and Lehning, 2013).

Demographic changes worldwide, including population aging and urbanization, draw attention to processes of well-being in older age, linked to active aging, community engagement, and life satisfaction. The sense of community can have implications for identifying best practices and sustainable interventions (Garon et al., 2014; Chan and Cao, 2015). Social connectedness and inclusion have been identified as major benefits of age-friendly initiatives (Menec et al., 2011; Emlet and Moceri, 2012). A positive social environment has been underscored in the context of facilitating community activities, learning education, volunteering, and employment as well as social support and providing home care assistance to enable older adults to age in place and retain independence (Steels, 2015).

Moving beyond infrastructure (e.g., housing, safety, transportation, health care, support services), Gonzales and Morrow-Howell (2009) have underscored the need to take into account productive engagement in older adults. At the same time, older adult’s understanding of the term “aging in place” has underscored a sense of attachment and feelings of security to both home and community as well as a sense of autonomy and independence through caring relationships and personal roles (Wiles et al., 2012). Interventions aiming at changing stereotypes of loneliness of old age can be particularly helpful as expectations related to loneliness have been found to be related to reported loneliness 8 years later (Pikhartova et al., 2015).

In addition to supporting older adults experiencing age-related challenges, there is also a need for population-level health efforts promoting preventive care (Greenfield et al., 2015). An aging population has immense social and economic consequences when countries are unprepared. The demand for services consumed by older adults (such as health care) will increase sharply while the base of employed workers and taxpayers to pay for this rising expenditure decreases in absolute and relative numbers. On a more positive note, a larger proportion of this age cohort is also likely to remain healthy as they age. More of them will thus be capable of continuing in employment or other forms of contribution to society for longer (Chan and Cao, 2015; Morrow-Howell et al., 2017). The psychological sense of community has been found to be a predictor of volunteerism in older adults (Omoto and Packard, 2016).

Volunteering by older adults can contribute to developing accessible and sustainable community interventions while at the same the time enhancing the well-being of the older adults who are volunteering (Au et al., 2016; Greenfield et al., 2016). For instance, older adult volunteers have been successfully trained to deliver an innovative telephone-assisted behavioral activation program to support dementia caregivers (Au, 2015; Au et al., 2015a,b,c, 2019c). In this program, senior volunteers helped to implement procedures like pleasant event scheduling and communication training. In addition, intergenerational partnerships as well as community–university partnership has also been examined in delivering care services to frail older adults (Au et al., 2015c).

At the same time, senior citizens themselves have reported various benefits through volunteering. These include enriching their lives, building relationships, enhancing self-concept, promoting health, as well as preparations for the rest of their lives (Chen, 2016). A peer education program targeted to promote quality use of medication in senior citizens has also identified that the life experience of the peer educators themselves played a significant role in contributing to peer learning. Moreover, the program also helps to overcome the sense of disempowerment as lay trainers helped to provide unique learning experiences in coaching others to be active partners (Klein et al., 2014).

Thus, there is increasing evidence that senior citizens can contribute effectively and also benefit from volunteering in community health programs. Perhaps, more importantly, volunteering can help to build a natural neighborhood network that can move beyond the concepts of “maintaining functional support” and “providing support to the needy” to the notion of promoting a more interdependent community.

The present findings support the mediating role of sense of community on the relationship between age-friendly domains and life satisfaction. This enhances our understanding of the processes involved in strengthening well-being in older adults with relevance to social connectedness and aging in place. However, several limitations in the present study must be acknowledged. First, this is a cross-sectional study. Future studies should consider longitudinal follow-up approaches, with attention to intervention components to improve SOC interventions and relationship building in the community (Christens, 2010; O’Connor, 2013).

Second, diversity of needs and possible systematic barriers to enhancing SOC should be identified. These include gender, socio-economic conditions, and caregiving responsibilities (Ohmer, 2010). Future work should identify how different types of neighborhood can contribute to older types with different socio-economic attribute such as family structure, income, and education (Yan et al., 2014; Park and Lee, 2017). While the needs of the very young old have been highlighted (Youmans, 1977), the present study has concentrated mainly on those who are physically able to go to community centers. Future studies should take into account those living with frailty and are more likely to be home-bound. Future investigations should also examine the possible differences in SOC between frail and non-frail community-dwelling older adults (Van Dijk et al., 2015).

Thirdly, in a recent study, achievement goal attainment was found to mediate between generativity (i.e., the concern for passing the good to the next generation) and positive emotion across three cultures (Au et al., 2019b). Thus, the building of SOC should also focus on creating opportunities for older adults to have opportunities to find fulfillment in intergeneration relationships. Moreover, the relationships between emotions and the more cognitive perceptions of life satisfaction need to be examined. Finally, the present study involves only self-report measures. Future studies should consider the use of objective measures such as health indices.

Despite these limitations, the present study has highlighted that AFC initiatives need to be promoted through promoting civic engagement opportunities through the creation of a conducive and supportive environment. Age-friendly urban planning can enhance life satisfaction, not only through physical infrastructure and facilities but also through opportunities for community engagement. Given the mediating role of sense of community, efforts to promote age-friendly urban planning and enhance life satisfaction among older adults should focus on expanding opportunities for building and strengthening community connections. These implications can have significance for global initiatives for promoting community networks and interdependence in regions where public services are challenged to meet the growing psycho-social needs associated with population aging.

## Data Availability Statement

The datasets generated for this study are available on request to the corresponding author.

## Ethics Statement

The studies involving human participants were reviewed and approved by the Research Office of The Hong Kong Polytechnic University. The patients/participants provided their written informed consent to participate in this study.

## Author Contributions

AA, DL, and HY oversaw the whole project in data collection and data management. AA, SL, and SC was responsible for developing the conceptual framework of the manuscript including data analysis and reporting. HC, AS, and GL contributed to and advised on the refinement of the conceptualization and data reporting with their expertise in population aging and age-friendliness.

## Conflict of Interest

The authors declare that the research was conducted in the absence of any commercial or financial relationships that could be construed as a potential conflict of interest.
